# Kratom alkaloid mitragynine: Inhibition of chemotherapy-induced peripheral neuropathy in mice is dependent on sex and active adrenergic and opioid receptors

**DOI:** 10.1016/j.ibneur.2022.08.007

**Published:** 2022-08-30

**Authors:** Daniel J. Farkas, Jeffery D. Foss, Sara Jane Ward, Scott M. Rawls

**Affiliations:** a Center for Substance Abuse Research, Lewis Katz School of Medicine, Temple University, 3500 North Broad Street, Philadelphia, PA 19140, USA; b Department of Pharmacology, Lewis Katz School of Medicine, Temple University, 3500 North Broad Street, Philadelphia, PA 19140, USA

**Keywords:** MG, mitragynine, CIPN, chemotherapy-induced peripheral neuropathy, Kratom, Mitragynine, Oxaliplatin, Neuropathic pain, Allodynia, Chemotherapy-induced peripheral neuropathy

## Abstract

Mitragynine (MG) is an alkaloid found in *Mitragyna speciosa* (kratom) that is used as an herbal remedy for pain relief and opioid withdrawal. MG acts at μ-opioid and α-adrenergic receptors in vitro*,* but the physiological relevance of this activity in the context of neuropathic pain remains unknown. The purpose of the present study was to characterize the effects of MG in a mouse model of chemotherapy-induced peripheral neuropathy (CIPN), and to investigate the potential impact of sex on MG’s therapeutic efficacy. Inhibition of oxaliplatin-induced mechanical hypersensitivity was measured following intraperitoneal administration of MG. Both male and female C57BL/6J mice were used to characterize potential sex-differences in MG’s therapeutic efficacy. Pharmacological mechanisms of MG were characterized through pretreatment with the opioid and adrenergic antagonists naltrexone, prazosin, yohimbine, and propranolol (1, 2.5, 5 mg/kg). Oxaliplatin produced significant mechanical allodynia of equal magnitude in both male and females, which was dose-dependently attenuated by repeated MG exposure. MG was more potent in males vs females, and the highest dose of MG (10 mg/kg) exhibited greater anti-allodynic efficacy in males. Mechanistically, activity at µ-opioid, α_1_- and α_2_-adrenergic receptors, but not β-adrenergic receptors contributed to the effects of MG against oxaliplatin-induced mechanical hypersensitivity. Repeated MG exposure significantly attenuated oxaliplatin-induced mechanical hypersensitivity with greater potency and efficacy in males, which has crucial implications in the context of individualized pain management. The opioid and adrenergic components of MG indicate that it shares pharmacological properties with clinical neuropathic pain treatments.

## Highlights

• Mitragynine significantly inhibits oxaliplatin-induced mechanical allodynia in mice.
• Mitragynine exhibited greater efficacy and potency in males compared to females.
• μ-opioid and α-adrenergic receptor activation contributes to mitragynine’s efficacy.

## 1 Introduction

Chemotherapy-induced peripheral neuropathy (CIPN) is a complex and severe chronic pain condition associated with chemotherapy exposure that is induced by damage to the peripheral nervous system. It is characterized by symptoms such as abnormal and spontaneous pain, hyperalgesia, allodynia, thermal hypersensitivity, numbness, and tingling predominantly afflicting the hands and feet. Acute symptoms of CIPN can develop within hours or days following chemotherapy infusion, whereas symptoms of CIPN persist in approximately 68 % of patients one month following their last chemotherapy treatment, with the ability to persist 6 months and beyond cessation of chemotherapy treatment in approximately 30 % of patients (Pachman et al., 2015, Seretny et al., 2014). CIPN prevalence is highly agent-specific, with the highest incidence rate (70–100 %) among individuals treated with platinum-based chemotherapeutics (Banach et al., 2016). Platinum-based agents such as oxaliplatin are predominantly used to treat colorectal cancer, in which an estimated 1.9 million new diagnoses were made worldwide in 2020 (Sung et al., 2021). Oxaliplatin produces acute, transient peripheral neuropathy in 65–98 % of patients hours following a single infusion at a dose range of 85–130 mg/m^2^, which can last 5–7 days (Gebremedhn et al., 2018, Leonard et al., 2005). Oxaliplatin-induced peripheral neuropathy is also capable of persisting chronically, lasting 24-months after discontinuation of treatment in 84 % of patients (Briani et al., 2014). However, reported incidence rates are variable and are largely associated with clinical factors such as differences in cumulative dose, timing of diagnosis, and variations in symptom presentation, severity, and persistence. Consequently, the exact mechanisms contributing to CIPN development and maintenance remain poorly understood.

In addition to the clinical sources of variability, demographic factors such as sex and gender can also profoundly contribute to the heterogeneity in the incidence of all forms of chronic pain. In general, CDC data suggest that the prevalence of both chronic pain and high-impact chronic pain is greater among US adult females compared to males (Redfield et al., 2018). Several studies have reported greater prevalence of numerous forms of neuropathic pain in females (Fillingim et al., 2009). More specifically, it has been shown that CIPN from oxaliplatin exposure occurs in comparable rates among males and females (Selvy et al., 2020, Shah et al., 2018). Yet, most preclinical studies focused on CIPN pathophysiology and behavior have been predominantly conducted in male subjects only. Consequently, it is of significant clinical interest to further investigate the impact of sex on comorbidities associated with chemotherapeutic exposure such as neuropathic pain in rodents.

The high efficacy of chemotherapeutic agents has led to an immense improvement in cancer survivorship regardless of demographics, with a projected 35 % further increase in 2022 (De Moor et al., 2013). However, this has led to a growing problem of cancer survivors suffering from chronic conditions and comorbidities that may persist long after completion of cancer treatment, such as neuropathic pain (Glare et al., 2014). Despite the rise in CIPN incidence, current treatments for neuropathic pain are limited in efficacy. The major drug classes implemented to treat neuropathic pain include antidepressants (tricyclic antidepressants [TCAs] and serotonin-norepinephrine reuptake inhibitors [SNRI’s]), anticonvulsants, and opioids (Cavalli et al., 2019). Compounds such as μ-opioid receptor agonists are not optimal for chronic use due to limited therapeutic benefit and increased risk of harm, despite being used by up 97 % of CIPN patients (Chou et al., 2015, Shah et al., 2018). Overall, the currently available treatments for chronic neuropathic pain are effective in <50 % of patients and are often accompanied by adverse effects during prolonged use (Finnerup et al., 2015). Therefore, there is a clear unmet need for novel treatment strategies for CIPN and other peripheral neuropathies that can replace and/or reduce the use of current treatment options.

In recent years there has been a growing interest in utilizing natural, plant-derived compounds as replacements for the treatment of chronic pain conditions, in hopes of achieving therapeutic efficacy while simultaneously minimizing adverse effects. Mitragyna speciosa (i.e*.,* kratom) has emerged as an alternative to traditional medical treatments for opioid and alcohol dependence (Kruegel and Grundmann, 2018). Kratom contains over 20 biologically active alkaloid constituents, with mitragynine (MG) being the most prevalent (Jansen and Prast, 1988, Raffa et al., 2013). Despite originating from a non-opium source, MG has a pharmacological profile that resembles traditional opioid compounds, as it displays affinity for μ-opioid receptors (7.2 nM) and is approximately ¼ as potent as morphine (Yue et al., 2018, Yusoff et al., 2017). MG has also been recently demonstrated to act at α-adrenergic receptor subtypes with low micromolar affinity (Obeng et al., 2020). These opioid and adrenergic properties of MG have physiological consequences, as previous reports have shown that MG is efficacious in rodent models of acute thermal pain, which are dependent upon the activation of both opioid and α_2_-adrenergic receptors (Kenjiro Matsumoto et al., 2005; Matsumoto et al., 1996; Takayama et al., 2002; Thongpradichote et al., 1998). Similarly, we recently reported that MG is effective in chronic pain models, as MG dose-dependently inhibits mechanical allodynia induced by oxaliplatin in rats, through mechanisms involving both opioid and α-adrenergic receptor activation (Foss et al., 2020). Only one other publication has demonstrated anti-allodynic effects of kratom alkaloids to date, however this was conducted in a nerve ligation model and focused on the effects of synthetic derivatives of kratom alkaloids (Kenjiro Matsumoto et al., 2014). Therefore, much remains unknown as to how MG mechanistically achieves its anti-allodynic effects against CIPN. Most of our understanding on kratom use patterns in humans originates from a male-bias literature, despite our understanding that nearly half of all kratom users in the US are female (Swogger et al., 2022). Furthermore, there is a serious lack of preclinical studies characterizing sex differences in the behavioral pharmacology of MG and other kratom alkaloids. Consequently, it is imperative to investigate the impact of sex on the pharmacology and therapeutic effects of kratom alkaloids, specifically in the context of pain.

Overall, MG is a plant-derived compound with a unique pharmacological profile that is suitable as a therapeutic alternative for the treatment of pain. The primary goal of the present study was to improve our understanding of the opioid and adrenergic components of MG pharmacology in a mouse model of oxaliplatin-induced mechanical hypersensitivity. Additionally, we aimed to determine the potential impact of sex on the pharmacological and therapeutic profile of MG in the context of CIPN, given that our current understanding of kratom pharmacology stems from a male bias literature. These findings will improve our understanding of MG pharmacology and further characterize MG’s therapeutic potential. Characterizing potential sex-differences in kratom alkaloid pharmacology will also reiterate the importance and relevance of individualized pain management in clinical settings.

## 2 Materials and methods

### 2.1 Animals

Adult male and female C57BL/6J mice (8–10 weeks of age) purchased from Taconic Biosciences (Rensselaer, NY) or Jackson Labs (Bar Harbor, ME) were group-housed under standard housing conditions on a 12-h light/dark cycle. C57BL/6J mice were chosen based on previous reports of their sensitivity to chemotherapy-induced nociceptive behaviors (Warncke et al., 2021). To account for sex as a biological variable, equal numbers of male and female mice were used for all experiments. All group sizes are n = 7–16 per experimental condition, with exact group numbers indicated in figure legends. Mice were allowed to acclimate to the animal facilities for five days prior to the beginning of experimentation. Food and water were available ad libitum throughout all experiments. Proper steps were taken to minimize pain and the number of mice used. All animals were euthanized by CO_2_ asphyxiation followed by cervical dislocation. All animal use procedures were conducted in accordance with the National Research Council and the National Academic Press publication for the Care and Use of Laboratory Animals (adopted for use by the National Institutes of Health), and all animal experimental procedures complied with the guidelines of the Temple University Institutional Animal Care and Use Committee (IACUC). All animal experiments reported adhere with the ARRIVE guidelines (du Sert et al., 2020).

### 2.2 Materials

Oxaliplatin was obtained from Temple University Hospital Pharmacy (Philadelphia, PA) as a 6 mg/ml concentration stock solution and diluted in sterile water (B. Braun Medical Inc., Irvine, CA). Mitragynine (MG) was obtained from Cayman Chemical Company (Ann Arbor, MI) with molecular weight and purity (>97.3 %) additionally confirmed by Dr. Allen Reitz (Fox Chase Chemical Diversity Center, Doylestown, PA). MG was dissolved in a vehicle of 20 % Tween 80 / 80 % sterile water. Naltrexone hydrochloride (NTX), prazosin hydrochloride (PRZ), and propranolol hydrochloride (PROP) were obtained from Sigma-Aldrich (St. Louis, MO) and dissolved in sterile water. Yohimbine hydrochloride (YOH) was purchased from Tocris Bioscience (Bristol, UK) and dissolved in sterile water. All drugs were administered intraperitoneally (IP) at a volume of 10 ml/kg.

### 2.3 Neuropathic pain experiments

Mice were placed in individual holder cages (Bioseb, Pinellas Park, FL) placed on top of a wire grid floor suspended 30 cm above the bench top and acclimated to the apparatus environment for 30 min before each testing session. Upon acclimation to the testing environment, mechanical allodynia was assessed using von Frey monofilaments of varying forces (0.07–2.0 g) applied to the plantar surface of the right hind paw. Monofilaments were held in a c-shape pattern on the plantar surface of the paw for six seconds as previously described (King et al., 2017, Ward et al., 2011, Ward et al., 2014). The smallest bending force that elicited a behavioral response was indicative of the animal’s mechanical threshold. Baseline mechanical sensitivity was assessed for each animal 30 min before oxaliplatin treatment (6 mg/kg). For dose response experiments, MG (1, 5, 10 mg/kg) or vehicle was administered once daily for six days. Behavioral assessment of mechanical allodynia was additionally measured on experimental days 2, 5, and 7. On days of simultaneous behavioral assessment and MG treatment, MG was administered 15 min following behavioral testing to circumvent potential acute analgesic effects of MG exposure. For antagonist experiments, mice were treated for six days with combinations of MG (10 mg/kg) plus vehicle, prazosin (1, 2.5, 5 mg/kg), yohimbine (1, 2.5, 5 mg/kg), propranolol (1, 2.5, 5 mg/kg), or naltrexone (1, 2.5, 5 mg/kg). Antagonists were administered 15 min before MG or vehicle treatment. Baseline mechanical sensitivity was measured prior to oxaliplatin exposure, and again on day 7. MG and antagonist doses are based on prior work (Choucair-Jaafar et al., 2009, Foss et al., 2020, Tanabe et al., 2005, Yalcin et al., 2009). Mice were randomly assigned to treatment groups across all studies presented.

#### 2.3.1 Experiment 1

To determine the role of sex on oxaliplatin-induced mechanical hypersensitivity, both males and females within the same cohort were treated with either vehicle solution (20 % Tween 80 / 80 % sterile water) or oxaliplatin (6 mg/kg) following baseline allodynia. Mice then received either vehicle solution or MG (10 mg/kg) once daily for the next six days, resulting in three experimental groups per sex: vehicle only, oxaliplatin + vehicle, and vehicle + MG. A vehicle + MG group was included for both sexes to determine whether repeated MG exposure would have inherent effects on mechanical hypersensitivity. Mechanical allodynia was additionally measured on days 2, 5, and 7 as described above. To detect potential sex-differences, males and females were run simultaneously to avoid inter-experimental variation.

#### 2.3.2 Experiment 2

The ability of MG to attenuate oxaliplatin-induced mechanical hypersensitivity was measured through repeated MG dosing following acute oxaliplatin exposure in separate cohorts of male and female mice. A dose range of MG (1, 5, 10 mg/kg) was tested against oxaliplatin in both sexes, resulting in four experimental groups per sex: oxaliplatin + vehicle solution, oxaliplatin + MG (1 mg/kg), oxaliplatin + MG (5 mg/kg), and oxaliplatin + MG (10 mg/kg). Animals were dosed with vehicle or MG for six consecutive days as previously described, and mechanical hypersensitivity was additionally measured on day 7. Male and female dose response studies were conducted separately.

#### 2.3.3 Experiment 3

To determine the impact of sex on the anti-allodynic efficacy of MG against oxaliplatin, both male and female mice within the same cohort were treated with a high dose of MG (10 mg/kg) for six consecutive days following acute oxaliplatin exposure, resulting in two experimental groups per sex: oxaliplatin + vehicle, and oxaliplatin + MG (10 mg/kg). Mechanical hypersensitivity was measured at baseline and again on day 7. To detect potential sex-differences, males and females were run simultaneously to avoid inter-experimental variation.

#### 2.3.4 Experiment 4

The pharmacological mechanism(s) contributing to the anti-allodynic efficacy of MG against oxaliplatin were determined through pretreatment with various pharmacological antagonists prior to high dose MG (10 mg/kg) exposure across days 1–6. To investigate adrenergic mechanisms of MG, mice were pretreated with either the α_1_-adrenergic antagonist prazosin, α_2_-adrenergic antagonist yohimbine, or β-adrenergic antagonist propranolol prior to MG. Naltrexone was administered prior to MG to investigate µ-opioid receptor mechanisms of MG. Based on the doses tested, each experiment contained six experimental groups: oxaliplatin + vehicle, oxaliplatin + MG, oxaliplatin + low dose antagonist + MG, oxaliplatin + middle dose antagonist + MG, oxaliplatin + high dose antagonist + MG, and oxaliplatin + high dose antagonist + vehicle. An oxaliplatin + high dose antagonist + vehicle treatment group was included to determine whether the chosen antagonists on their own would have any inherent effects on mechanical hypersensitivity. Mechanical hypersensitivity was measured at baseline and again on day 7. For all mechanistic studies, male and female studies were conducted separately.

### 2.4 Data and statistical analysis

The data and statistical analysis of all present studies comply with the recommendations on experimental design and analysis in pharmacology (Curtis et al., 2018). All behavioral data are depicted as a function of mean mechanical threshold (g) corresponding to filament thickness. Mechanical allodynia conducted in Experiment 1 was analyzed by three-way ANOVA (time x treatment x sex) and Tukey’s multiple comparisons test. Body weights are depicted as mean percent change in body weight and were analyzed by two-way ANOVA (sex x treatment) and Tukey’s multiple comparisons test. In experiments 2–4, the terminal behavioral timepoint of experimental day 7 was used to represent behavioral data based on our initial observations of peak oxaliplatin-induced mechanical allodynia on day 7 observed in Experiment 1 and as previously described (Foss et al., 2020). To detect sex differences, a two-way ANOVA was performed (sex x treatment) to analyze data sets where behavioral analysis was conducted on males and females simultaneously within the same cohort (Experiments 1 & 3). Tukey’s multiple comparisons test was performed to detect individual differences between groups in the instance of significance by two-way ANOVA. One-way ANOVA (treatment) was performed to analyze data sets in which behavior in males and females was conducted in separate experiments. Dunnett’s multiple comparisons test was performed to detect individual differences between groups in the instance of significance by one-way ANOVA in dose response studies (Experiment 2), and Bonferroni’s multiple comparisons test was performed for antagonist studies (Experiment 4). In all instances, statistical significance was set at p < 0.05. All data are plotted as mean ± SEM. Across all studies, outliers were determined using the exclusion criteria of a Z-score = 2. All statistical analyses were conducted using GraphPad Prism 9.

## 3 Results

### 3.1 Experiment 1

#### 3.1.1 Oxaliplatin produced mechanical hypersensitivity of approximately equal severity in male and female mice

**Fig. 1:**
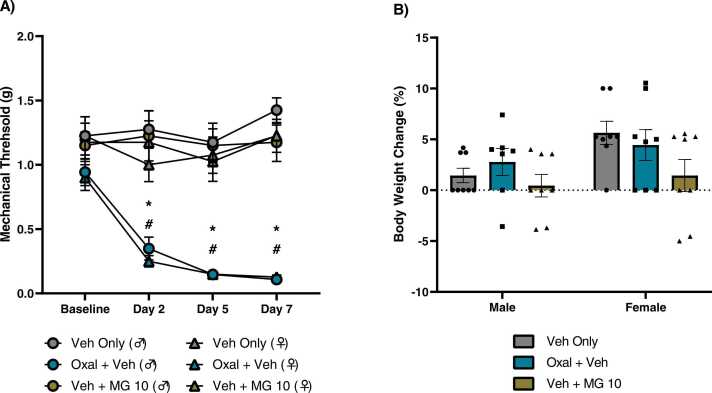
Effect of acute oxaliplatin exposure on mechanical hypersensitivity and body weight in male and female mice. Baseline mechanical sensitivity was measured prior to treatment with a single injection of oxaliplatin or vehicle in both males and females. Animals then received either MG (10 mg/kg) or vehicle for six consecutive days. Mechanical sensitivity was additionally measured on days 2, 5, and 7. Animals were weighed prior to measuring baseline mechanical sensitivity, and again on day 7. A) Time course of mechanical hypersensitivity. A single outlier was determined at baseline for male oxal + veh group, and this animal was removed from the study. Therefore, n = 7 for male oxal + veh, n = 8 for all other groups. * indicates significance of male veh only compared to male oxal + veh. # indicates significance of female veh only compares to female oxal + veh. B) Percent change in animal body weight at day 7 compared to baseline**.** P < 0.05. Data plotted as mean ± SEM.

Three-way ANOVA revealed significant main effects of time [F _(3,28)_ = 10.27, P < 0.0001], treatment [F _(1,28)_ = 198.9, P < 0.0001], time x treatment [F _(3,28)_ = 10.48, P < 0.0001], but not sex (Fig. 1A). Post-hoc analysis did not reveal any significant differences in baseline threshold values across all treatment groups. Oxaliplatin produced significant mechanical hypersensitivity in both sexes across days 2 (male P < 0.0001, female P < 0.001), 5 (male and female P < 0.0001), and 7 (male and female P < 0.0001). No significant differences were detected between oxaliplatin-treated males and females across days 2, 5, and 7. Both oxaliplatin-treated males and females exhibited peak mechanical hypersensitivity on day 7, with mean mechanical threshold values of 0.10 g and 0.12 g, respectively. In both sexes, vehicle + MG controls did not exhibit any inherent sensitivity to the behavioral paradigm, suggesting that high doses of MG alone are not sufficient to deviate mean mechanical threshold values from baseline.

#### 3.1.2 Acute oxaliplatin nor repeated MG exposure did not significantly impact body weight

Body weights were recorded prior to baseline allodynia and again on day 7 to determine the effects of acute oxaliplatin and repeated MG exposure on weight changes in both males and females. Two-way ANOVA revealed a significant main effect on sex [F _(1,41)_ = 4.905, P < 0.05] (Fig. 1B). Post-hoc analysis did not reveal any significant differences in percent change in body weight across all treatment groups.

### 3.2 Experiment 2

#### 3.2.1 MG inhibited oxaliplatin-induced mechanical allodynia in both male and female mice

**Fig. 2:**
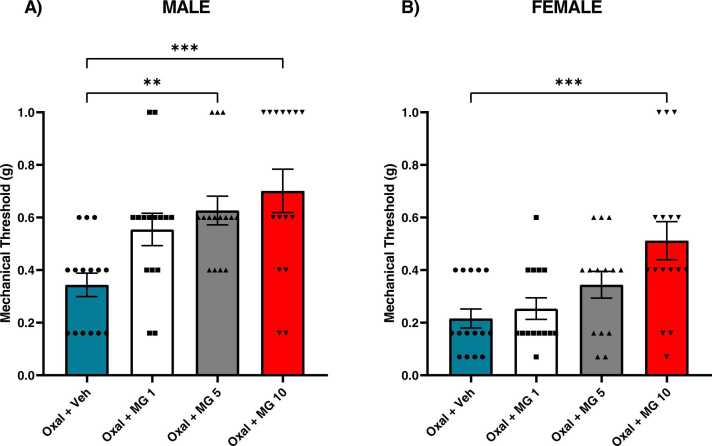
Effect of repeated MG exposure on oxaliplatin-induced mechanical hypersensitivity in male and female mice. Mice were treated with vehicle or MG (1–10 mg/kg) for six consecutive days following a single oxaliplatin injection. Baseline mechanical sensitivity was measured prior to oxaliplatin injection, and again on day 7. Data plotted as day 7 timepoint. A) Male MG dose response. A single outlier was determined in each MG treatment group at baseline, and these animals were removed from the study. Therefore, n = 15 for all three MG-treated groups and n = 16 for oxal + veh group. B) Female MG dose response. N = 16 for all treatment groups. P < 0.05. Data plotted as mean ± SEM.

To characterize the therapeutic efficacy of MG against oxaliplatin-induced mechanical hypersensitivity, mice were treated with a dose range of MG following oxaliplatin exposure. In males, one-way ANOVA revealed a significant main effect of treatment [F _(3,56)_ = 6.071 = P < 0.01] (Fig. 2A). Mean mechanical thresholds were significantly higher in males treated with median (5 mg/kg) (P < 0.01) and high (10 mg/kg) (P < 0.001) doses of MG compared to vehicle-treated controls. The low dose of MG (1 mg/kg) produced trending, but statistically non-significant anti-allodynic effects (P = 0.0536). In females, one-way ANOVA revealed a significant main effect of treatment [F _(3,55)_ = 6.394, P < 0.001] (Fig. 2B). Significant anti-allodynic effects were produced with 10 mg/kg of MG only (P < 0.001).

### 3.3 Experiment 3

#### 3.3.1 MG produced sex-dependent effects against oxaliplatin-induced mechanical hypersensitivity

**Fig. 3:**
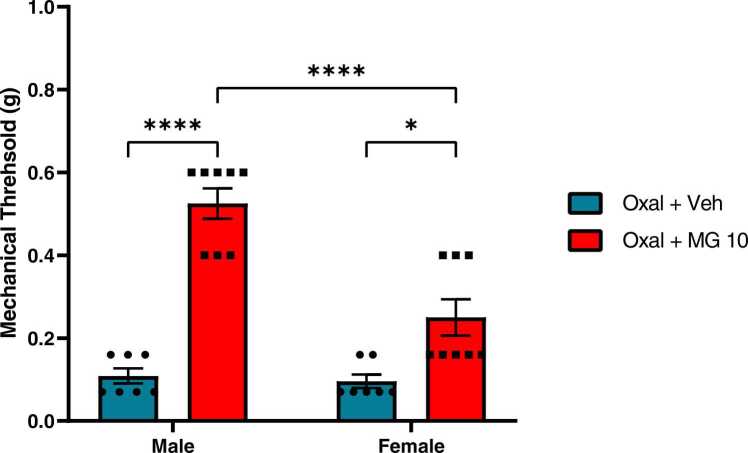
Effect of sex on the therapeutic effects of MG against oxaliplatin-induced mechanical hypersensitivity. Mice were treated with MG (10 mg/kg) or vehicle for six consecutive days following a single oxaliplatin injection. Baseline mechanical sensitivity was measured prior to oxaliplatin injection, and again on day 7. Data plotted as day 7 timepoint. N = 8 for male treatment groups. A single outlier was determined at baseline for female oxal + veh group, and this animal was removed from the study. Therefore, n = 7 for oxal + veh females and n = 8 for MG-treated females. P < 0.05. Data plotted as mean ± SEM.

To determine whether MG inhibited mechanical hypersensitivity in a sex-dependent manner, we exposed both males and females to oxaliplatin followed by vehicle or MG (10 mg/kg). Two-way ANOVA revealed a significant main effect of treatment [F _(1,26)_ = 75.83, P < 0.0001], sex [F _(1,26)_ = 19.29, P < 0.001], and treatment x sex [F _(1,26)_ = 16.00, P < 0.001] (Fig. 3). MG produced significant anti-allodynic effects in both males and females compared to their respective vehicle-treated controls (male P < 0.0001, female P < 0.05). Although no significant differences were detected between male and female controls, mean mechanical threshold values were significantly higher in MG-treated males compared to MG-treated females (P < 0.0001).

### 3.4 Experiment 4

#### 3.4.1 µ-opioid receptor activation contributes to the anti-allodynic efficacy of MG in males, but not females

**Fig. 4:**
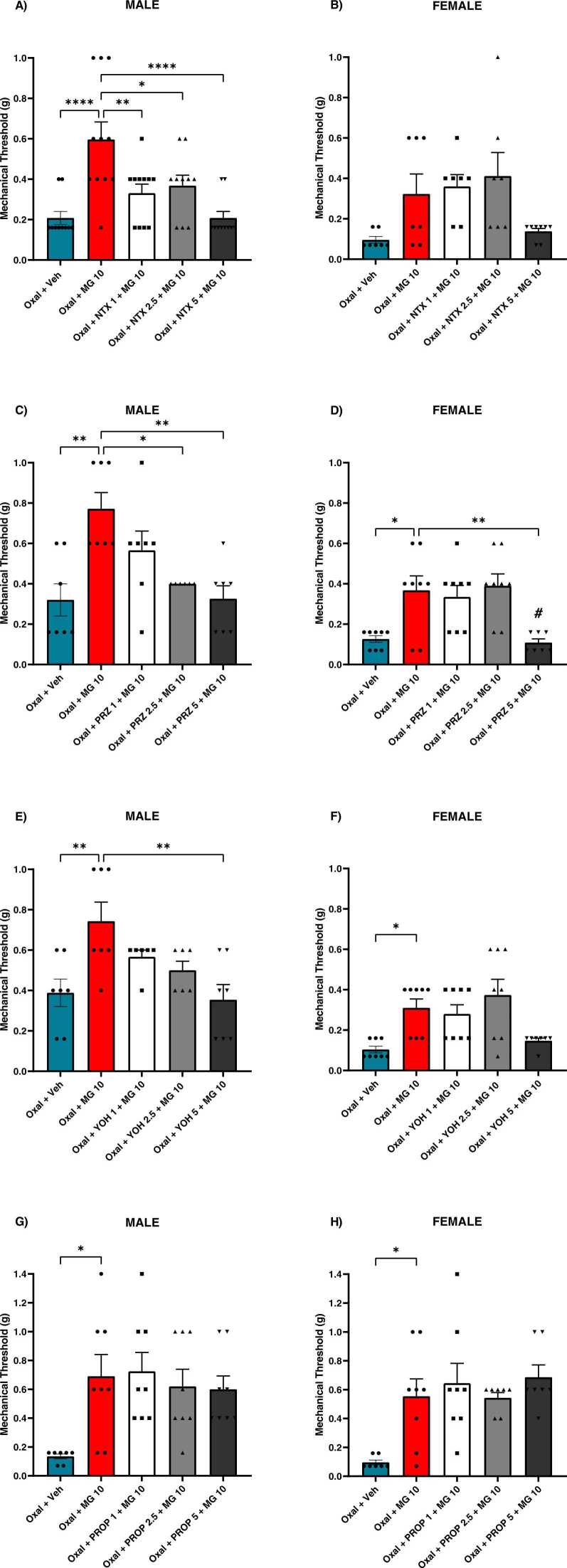
Effects of opioid and adrenergic receptor activation on the anti-allodynic efficacy of MG against oxaliplatin in male and female mice. Baseline mechanical sensitivity was measured prior to oxaliplatin injection, and again on day 7. Then for six consecutive days, mice were pretreated with either vehicle, naltrexone, prazosin, yohimbine, or propranolol (1–5 mg/kg) 15 min prior to injection of either MG (10 mg/kg) or vehicle. A) Male naltrexone pretreatment. A single outlier was determined at baseline in NTX 2.5 group, therefore n = 10–11. B) Female naltrexone pretreatment. A single outlier was determined at baseline in NTX 2.5 group, therefore n = 7–8. C) Male prazosin pretreatment. N = 8. D) Female prazosin pretreatment. N = 8. E) Male yohimbine pretreatment. N = 7. F) Female yohimbine pretreatment. N = 8. G) Male propranolol pretreatment. N = 8. H) Female propranolol pretreatment. N = 8. Data plotted as day 7 timepoint. * indicates significance from oxal + veh. # indicates significance from oxal + MG 10. P < 0.05. Data plotted as mean ± SEM.

To characterize the potential opioid component of MG’s therapeutic profile against oxaliplatin-induced mechanical hypersensitivity, mice were pretreated with µ-opioid receptor antagonist naltrexone prior to each MG injection. In males, one-way ANOVA revealed a significant main effect of treatment [F _(4,47)_ = 8.566, P < 0.0001] (Fig. 4A). Oxal + MG 10 males exhibited significantly higher mean mechanical thresholds compared to oxaliplatin alone (P < 0.0001). The anti-allodynic effect produced by MG was suppressed by naltrexone at 1 mg/kg (P < 0.01), 2.5 mg/kg (P < 0.05), and 5 mg/kg (P < 0.0001). In females, one-way ANOVA revealed a significant main effect of treatment [F _(4,31)_ = 3.818, P < 0.05] (Fig. 4B). However, MG-treated females were not significantly different than controls, nor were any naltrexone-pretreated animals significantly different than those treated with MG alone.

#### 3.4.2 α-adrenergic receptor activation contributes to the anti-allodynic efficacy of MG in both males and females

To improve our understanding of the adrenergic components of MG pharmacology, mice were pretreated with the adrenergic antagonists prazosin, yohimbine, or propranolol prior to MG. In prazosin-treated males, one-way ANOVA revealed a significant main effect [F _(4,29)_ = 6.793, P < 0.001] (Fig. 4C). Treatment with MG alone significantly inhibited oxaliplatin-induced mechanical hypersensitivity (P < 0.01). Prazosin pretreatment significantly suppressed the anti-allodynic effect of MG at 2.5 mg/kg (P < 0.05) and 5 mg/kg (P < 0.01). One-way ANOVA also revealed a significant main effect in prazosin-treated females [F _(4,34)_ = 7.037, P < 0.001] (Fig. 4D). MG alone produced significant anti-allodynic effects (P < 0.05), however prazosin pretreatment significantly suppressed the effects of MG at 5 mg/kg only (P < 0.01).

In yohimbine-treated males, one-way ANOVA revealed a significant main effect [F _(4,28)_ = 5.212, P < 0.01] (Fig. 4E). MG alone significantly inhibited mechanical hypersensitivity produced by oxaliplatin alone (P < 0.01), which was significantly suppressed by 5 mg/kg yohimbine pretreatment (P < 0.01). One-way ANOVA also revealed a significant main effect in yohimbine-treated females [F _(4,34)_ = 5.864, P < 0.01] (Fig. 4F). MG significantly improved mean mechanical thresholds compared to controls (P < 0.05). However, across all doses tested, yohimbine-pretreated females were not significantly different from MG alone. Lastly, one-way ANOVA revealed a significant main effect in both propranolol-treated males [F _(4,34)_ = 4.015, P < 0.01] (Fig. 4 G) and females [F _(4,32)_ = 5.635, P < 0.01] (Fig. 4H). MG produced significant anti-allodynic effects in both males and females (P < 0.05). However, no dose of propranolol pretreatment tested produced significantly different threshold values than MG alone in either sex.

## 4 Discussion

The present experiments characterized the mechanisms contributing to the anti-allodynic efficacy of MG in a mouse model of CIPN and determined the impact of sex on the therapeutic efficacy of MG against oxaliplatin-induced mechanical hypersensitivity. We first determined whether males or females displayed differences in mechanical sensitivity to oxaliplatin alone or displayed any inherent sensitivities to the behavioral assay. Acute oxaliplatin exposure produced robust mechanical allodynia in both male and female mice as early as 48 h following oxaliplatin treatment. Mean thresholds of oxaliplatin-naïve mice did not differ between sexes across all timepoints, suggesting no inherent effect of sex on sensitivity to the assay. Oxaliplatin alone produced significant mechanical allodynia in both sexes starting at day 2 and peaking at day 7. Mean mechanical threshold values were nearly identical between males and females across days 2–7, suggesting no differences in the allodynia inflicted by oxaliplatin. We also demonstrated that neither oxaliplatin nor MG exposure significantly impacted weight changes in either sex. A significant sex effect was found by two-way ANOVA; however, this is likely a consequence of transforming the data as percent change. Given that females weighed significantly less than males at baseline, changes in female body weight had greater influence on percent change than males. When calculating the absolute change in body weight in grams, no significant differences were detected (Supplementary Table 1). Our initial behavioral findings agree with the previous work of others, which demonstrates no effect of sex on oxaliplatin-induced mechanical hypersensitivity at both 0.3 and 30 mg/kg cumulative doses of oxaliplatin (Warncke et al., 2021). In contrast, they reported significant decreases in body weight in both sexes compared to controls as early as one week following the first oxaliplatin injection.

MG prevented the development of oxaliplatin-induced mechanical hypersensitivity in both male and female C57BL/6J mice. In males, MG significantly inhibited oxaliplatin-induced hypersensitivity at doses of 5 mg/kg and 10 mg/kg. These findings mirror what our laboratory has previously observed in rats treated with oxaliplatin, suggesting limited species specificity (Foss et al., 2020). In contrast, in females MG produced significant anti-allodynic effects at only the high dose of 10 mg/kg. These results suggest a potential disparity in the potency of MG between male and female mice. To confirm this, we compared this equi-effective dose of 10 mg/kg of MG in both sexes simultaneously to negate inter-experimental variability. We subsequently replicated that 10 mg/kg of MG produced significant anti-allodynic effects in both sexes, and that no sex-dependent effects on mechanical hypersensitivity were produced by oxaliplatin alone. However, we found that 10 mg/kg of MG produced significantly higher mechanical threshold values in males compared to females, suggestive of sex-differences in the therapeutic efficacy of MG.

Lastly, we characterized the opioid and adrenergic components of MG in a mouse model of CIPN across sexes. Naltrexone pretreatment significantly blocked the anti-allodynic effect of MG alone across all three doses in males, indicating a µ-opioid component of MG in CIPN. These results are consistent with our previous findings in addition to others in acute pain models (Foss et al., 2020; Matsumoto et al., 2004, Matsumoto et al., 2006, Matsumoto et al., 2014; Thongpradichote et al., 1998). However, no dose of naltrexone tested significantly blocked that of MG alone in females, suggesting differences in the opioid components of MG between sexes. Interestingly, prazosin was the only antagonist tested that inhibited MG’s therapeutic effect in both sexes. This suggests a central role of α_1_-adrenergic receptor activation in MG pharmacology, adding to previous findings demonstrating micromolar affinity of MG for various α_1_-adrenergic receptor subtypes (Obeng et al., 2020). Like naltrexone, yohimbine pretreatment significantly blocked MG’s effects in males only. α_2_-adrenergic properties of MG have been previously described both in vitro and in vivo (Boyer et al., 2008; Matsumoto et al., 1996; Obeng et al., 2020). However, given the low receptor subtype specificity at which yohimbine antagonizes α_2_-adrenergic receptors, we are unable to conclude which α_2_-adrenergic receptor subtypes may be driving the adrenergic components of MG’s therapeutic efficacy against CIPN. Propranolol was the only antagonist tested in which its pretreatment did not result in significant inhibition of MG’s anti-allodynic effect in either sex, suggesting that β-adrenergic receptors likely do not contribute to the development and maintenance of CIPN. This has been previously suggested, as spinal application of the β-adrenergic receptor agonist isoprenaline does not alleviate oxaliplatin-induced allodynia (Choi et al., 2017). However, testing a wider dose range of propranolol may be warranted, as doses up to 20 mg/kg have been shown to potentiate morphine antinociception (Afify and Andijani, 2017).

The dual μ-opioid and α-adrenergic components of MG raises the question of whether these receptor systems are activated to achieve therapeutic efficacy through independent mechanisms, or perhaps acting in a synergistic manner through a common downstream pathway. Both opioid receptors and specifically α_2_-adrenergic receptors are predominantly coupled with Gα_i/o_ subunits, which suggests a possible common downstream mechanism. Numerous studies have reported that protein kinase C (PKC), but not protein kinase A (PKA) inhibitors administered intrathecally are sufficient to block the synergistic interactions between opioids and α-adrenergic agonists without disrupting the antinociceptive properties of either compound alone, indicative of a common downstream mechanism (Chabot-Doré et al., 2015). Consequently, studies characterizing the effects of MG exposure on spinal PKC signaling are warranted. Interestingly, evidence suggests opioid-adrenergic synergistic interactions are limited to the spinal cord, despite abundant receptor co-expression in the locus coeruleus (Stone and Wilcox, 2004). However, the distribution patterns of μ-opioid and α-adrenergic receptors at the level of the spinal cord are diverse, complicating the ability to envision a synergistic mechanism of action. α_2A_-adrenergic receptors inhibit norepinephrine release through a presynaptic autoreceptor function on descending noradrenergic terminals, while α_2 C_-adrenergic receptors primarily act on spinal GABAergic and cholinergic interneurons (Gilsbach et al., 2009, Olave and Maxwell, 2003). Contrarily, μ-opioid receptors populate primary afferents (Beaudry et al., 2011). Overall, this distribution pattern suggests that MG may achieve therapeutic efficacy through modulating active opioid and adrenergic receptors that are in series with one another within spinal pathways.

Differences in drug pharmacokinetics can often provide insight into differences in the therapeutic efficacy of pharmacological agents. In turn, biological sex can play a significant role in the therapeutic efficacy of multiple classes of drugs such as opioids and antidepressants (Fullerton et al., 2018, LeGates et al., 2019). Given the pharmacological similarities between MG and both opioids and antidepressants, this serves as a plausible explanation for the observed sex-differences in the therapeutic efficacy of MG in CIPN. Few preclinical studies characterizing the pharmacokinetics of kratom alkaloids exist, with the majority having been conducted exclusively in a single sex (Avery et al., 2019, Kamble et al., 2021, Kong et al., 2017), with the exception of a recent study which reported that higher concentrations of the potent MG metabolite, 7-hydroxymitragynine, were obtained in male mice compared to females (Berthold et al., 2022). 7-hydroxymitragynine has much higher affinity at μ-opioid receptors than MG and produces antinociception with 40-fold greater potency than MG (Matsumoto et al., 2004, Takayama et al., 2002, Váradi et al., 2016). However, the contribution of 7-hydroxymitragynine has yet to be explored in the context of CIPN. Consequently, it is possible that sex differences in MG pharmacokinetics may underlie the observed sex differences in efficacy in a model of CIPN.

One limitation of the data presented is the principle of basement threshold values corresponding to the thickness and force of the von Frey monofilaments. Consequently, this complicates the ability to identify differences in mechanical hypersensitivity between animals both exhibiting mechanical allodynia. Additionally, the interpretation of the presented studies is limited by the systemic delivery of MG across all experiments, as kratom is traditionally consumed through oral route of administration in tea or pill form. The analgesic effects of oral administration of kratom alkaloids have been described in models of both acute and chronic pain (Kenjiro Matsumoto et al., 2004; Raffa et al., 2013). Specifically, oral administration of the synthetic MG derivative MGM-16 has been to shown to significantly improve mechanical thresholds in a mouse model of sciatic nerve ligation (Matsumoto et al., 2014). However, the therapeutic efficacy of oral kratom alkaloid consumption in rodent models of CIPN has not yet been determined.

In summary, the present studies demonstrated that the kratom alkaloid MG reduced oxaliplatin-induced mechanical hypersensitivity in mice through both α-adrenergic and µ-opioid, but not β-adrenergic mechanisms. Secondly, these studies demonstrated the disparity in the anti-allodynic efficacy of MG between male and female mice. Equivalent doses of MG produced significantly greater anti-allodynic effects in male mice, suggesting that higher doses of MG are required to achieve comparable therapeutic effects in female mice. Future studies will focus on identifying spinal and/or supraspinal sites of action of MG through intracerebroventricular (i.c.v.) and intrathecal (i.t.) administration of MG. Given the dual adrenergic and opioid pharmacology of MG, it is imperative to compare the effects of MG alongside or in combination with drugs that are clinically used to treat forms of neuropathic pain such as tramadol, tapentadol, duloxetine and venlafaxine. These studies would help determine whether MG would be more suitable as a standalone treatment option or adjunct therapy for CIPN. The efficacy of MG has also yet to be demonstrated in alternative CIPN models such as paclitaxel or cisplatin. Lastly, the therapeutic effects of MG will be tested in alternative chronic pain models such as diabetic neuropathy.

## CRediT authorship contribution statement

Daniel Farkas and Jeffery Foss conducted behavioral testing. Sara Jane Ward and Scott Rawls contributed to experimental design, data analysis and interpretation of the results. Daniel Farkas prepared the manuscript, which was circulated to all contributing authors for comments, edits, and approval.

## Conflict of Interest

All authors declare they have no conflicts of interest to report.
