# Supplementary material for: Kratom alkaloid mitragynine: Inhibition of chemotherapy-induced peripheral neuropathy in mice is dependent on sex and active adrenergic and opioid receptors
Source: IBRO Neurosci Rep. 2022 Aug 30;13:198–206. doi: 10.1016/j.ibneur.2022.08.007 (PMC9459671; doi:10.1016/j.ibneur.2022.08.007)
Supplement: Supplementary file 1 — Supplementary material [file mmc1.docx]

Supplementary Information


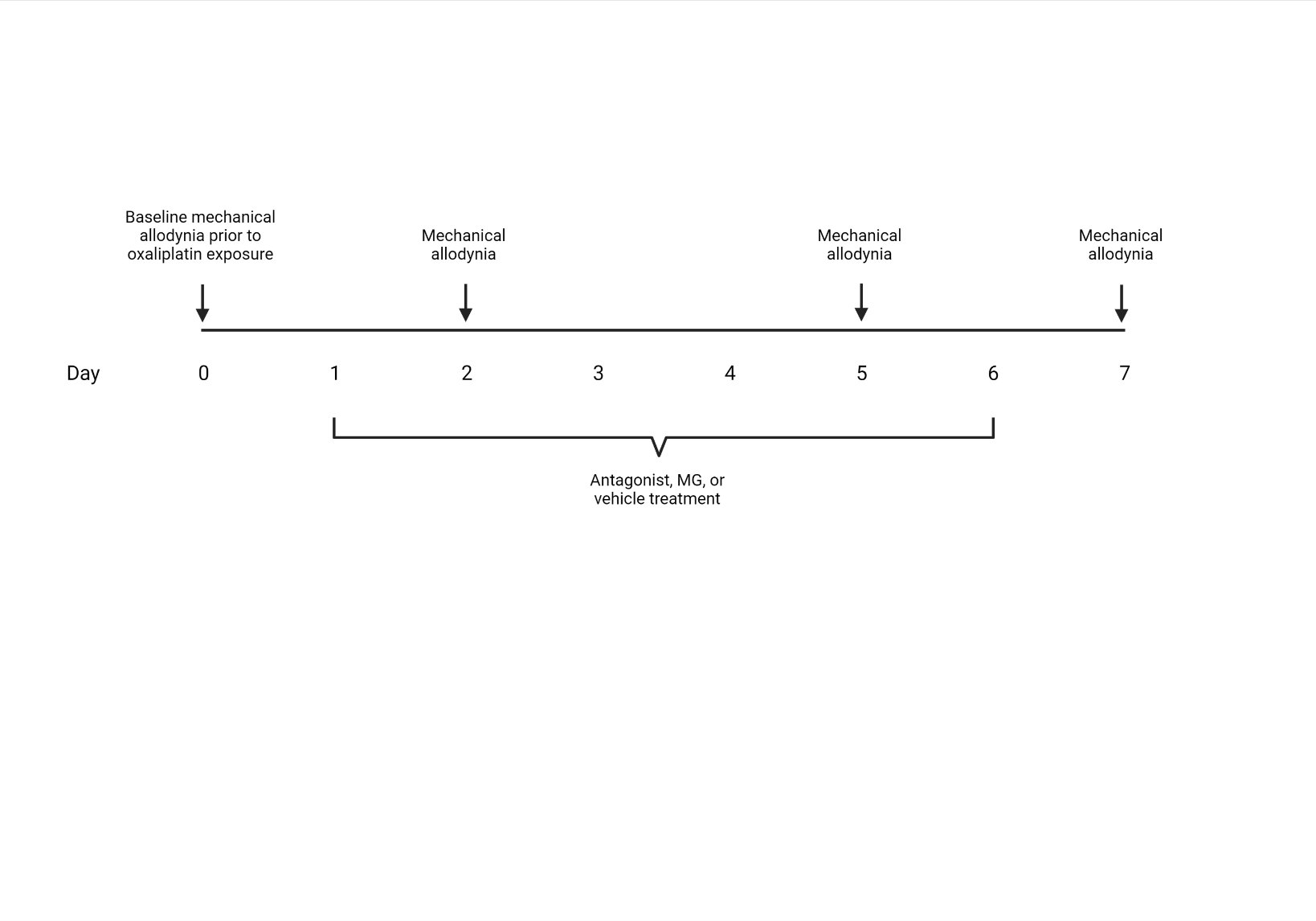
**Figure S1. Experimental timeline of behavioral assessment of mechanical allodynia.**

In Experiment 1, mechanical hypersensitivity was measured with von Frey monofilaments at day 0 (baseline), and again on days 2, 5, and 7. In Experiments 2-4, mechanical hypersensitivity was measured at days 0 and 7.

**Experiment 1.**

**Table S1. Mean animal weights and absolute change in body weight by gram.**

| **Treatment Group** | **Mean Baseline Weight (g)** | **Mean Day 7 Weight (g)** | **Mean Body Weight Change (g)** |
| --- | --- | --- | --- |
| Veh Only (♂) | 25.75 | 26.13 | +0.38 |
| Oxal + Veh (♂) | 26.29 | 27 | +0.71 |
| Veh + MG 10 (♂) | 27 | 27.13 | +0.13 |
| Veh Only (♀) | 20.13 | 21.25 | +1.12 |
| Oxal + Veh (♀) | 20.13 | 21 | +0.87 |
| Veh + MG 10 (♀) | 19.75 | 20 | +0.25 |

Two-way ANOVA did not reveal significant main effects of sex [F_(1,2)_ = 2.880, P = 0.2318] or treatment [F_(2,2)_ = 3.739, P = 0.2110] on absolute changes in mean body weight.
